# Eosinophilic Granulomatosis With Polyangiitis With Extensive Cutaneous Involvement

**DOI:** 10.7759/cureus.18581

**Published:** 2021-10-07

**Authors:** Cristina Silva, Sara Freitas, Ana Costa, Glória Alves, Jorge Cotter

**Affiliations:** 1 Internal Medicine, Hospital Senhora da Oliveira, Guimarães, PRT

**Keywords:** autoimmunity, skin lesions, vasculitis, churg-strauss syndrome, eosinophilic granulomatosis with polyangiitis

## Abstract

Eosinophilic granulomatosis with polyangiitis (EGPA) is a rare systemic vasculitis. This report describes the case of a 68-year-old female that showed up at the emergency department with extensive haemorrhagic bullous lesions, affecting elbows, the dorsal side of hands, feet and knees, with loss of tissue and necrotic areas. The evaluation led to the diagnosis of antineutrophil cytoplasmic antibody-positive EGPA with multisystem involvement: cutaneous, pulmonary, renal, intestinal and peripheral and central nervous system. She received corticosteroids and intravenous immunoglobulin. She developed multiple infectious complications with multidrug-resistant bacteria. Two months after the diagnosis, the patient had no respiratory or gastrointestinal signs or symptoms, and the proteinuria was mild. Yet, she maintained extensive ulcers and was suffering from disabling dysesthesias. After the resolution of all infections, we decided to start rituximab. She was also submitted to excisional debridement and heterologous graft repair and later to autologous graft repair of elbows and feet. She had a good clinical response with complete healing of the wounds. This case intends to illustrate a serious form of EGPA, with severe multisystem involvement that resulted in great morbidity. It was a clinical challenge to balance the need for immunosuppressive therapy with the high infectious risk of the patient. Nonetheless, we considered that disease control was fundamental to skin recovery, better physical rehabilitation and better quality of life.

## Introduction

Eosinophilic granulomatosis with polyangiitis (EGPA) is a rare systemic vasculitis. It has an annual incidence of 0.9-2.4 per million and an annual prevalence of 10.7-17.8 per million. Median age at onset is 49-59 years, but it can occur at any age [1]. The female-to-male ratio is about 1.2:1 [2].

It is characterized by adult-onset asthma, eosinophilic inflammation, extravascular granuloma formation, and necrotizing vasculitis, predominantly affecting small and medium-sized vessels [1-4]. Three stages of the disease have been described. First, there is an allergic stage with asthma and rhinosinusitis. Then, there is an eosinophilic stage with tissue and peripheral eosinophilia. At last, the vasculitic stage is marked by necrotizing inflammation of medium and small vessels, extravascular granulomatosis, and end-organ damage [4-6].

Peripheral nerve and paranasal sinuses are the most frequently involved organs [1]. The lung is the third most frequently affected organ and often dominates the clinical picture with asthma attacks and migrating pulmonary infiltrates [1-2]. About 39-52% of the patients develop skin abnormalities like palpable purpuras, hemorrhagic bullae, and subcutaneous papules and nodules [1,3]. Kidney involvement is less common [1-2]. Heart, ocular and gastrointestinal involvements are rarer but can also be seen [1-2].

Antineutrophil cytoplasmic antibodies (ANCA) are positive in 30-47% of EGPA patients and the subtype more frequently found is myeloperoxidase-ANCA [1]. Clinical differences associated with ANCA status have been described. Mononeuritis multiplex, glomerular nephritis and alveolar hemorrhage are more frequent in ANCA-positive patients, whereas heart involvement is more frequent in ANCA-negative patients [1,3,5-6].

For remission-induction of new-onset of organ- or life-threatening disease, it is recommended a combination of glucocorticoids and either cyclophosphamide or rituximab. For non-organ-threatening EGPA, it is recommended a combination of glucocorticoids and either methotrexate or mycophenolate mofetil. Life-threatening conditions, as rapidly progressive glomerulonephritis or alveolar hemorrhage, may require plasma exchange. For remission-maintenance of EGPA, it is recommended a combination of low-dose glucocorticoids and either azathioprine, rituximab, methotrexate or mycophenolate mofetil [7-8].

## Case presentation

Emergency Department evaluation

A 68-year-old female went to the emergency department (ED) because of neck pain and arthralgia of shoulders, left hip and left foot that started three days before. Basic workup showed leukocytosis (13.2x10^3/µL) with eosinophilia (53.5%), C-reactive protein 107 mg/L (N<3 mg/L) and leukocyturia (414 cells/µL). She was discharged with a diagnosis of osteoarthritis and cystitis, medicated with etoricoxib 90 mg once a day orally and ciprofloxacin 500 mg two times a day (bid). Three days later, she returned to the ED because of pain in the left superior limb and bilateral hand dysesthesia. She received 75 mg of intramuscular diclofenac and was discharged with paracetamol/thiocolchicoside 1000/4 mg three times a day (tid). Another three days later, she went to the ED because of painful cutaneous lesions. She had extensive well-defined purpuric patches with extensive ulceration in elbows, bullae in the dorsal face of feet and posterior face of both legs, necrotic areas in the dorsal side of hands, and oral ulcers. Laboratory tests showed leukocytosis (15.4x10^3/µL) with eosinophilia (8.3x10^3/µL), C-reactive protein 522 mg/L (N<3 mg/L) and stage 3 acute kidney injury (creatinine 3.81 mg/dL vs 0.84 mg/dL seven days before). A skin biopsy was performed, and she was transferred to a Burn Intensive Care Unit in another hospital. At that time, the main diagnostic hypothesis was Stevens-Johnson syndrome.

Management in the Burn Intensive Care Unit

The subsequent evaluation in the Burn Intensive Care Unit led to the diagnosis of EGPA on day five of hospital stay (D5). The patient had asthma diagnosed at age 35 and was medicated with fluticasone/salmeterol inhaler 500/50 μg bid. She also had chronic rhinitis and nasal polyposis, medicated with nasal fluticasone 50 μg bid. When blood counts of the last year were analyzed, persistent eosinophilia was evident, with a total eosinophil count varying from 1.6-5.2x10^3/µL. The total eosinophil count was now 8.3x10^3/µL, as previously mentioned. ​​​​The skin biopsy showed extensive lesions of neutrophilic and eosinophilic vasculitis affecting small and medium-sized blood vessels. Blood tests were positive for myeloperoxidase-ANCA.

She had multisystem involvement by the disease as follows: skin: extensive areas of epidermolysis and necrosis; kidney: acute kidney injury (maximum creatinine of 3.81 mg/dL) and nephrotic proteinuria (5 g/24h); nervous system: peripheral neuropathy and cerebral vasculitis (right central facial palsy and paresis of the right superior limb observed on D1); gastrointestinal: ischemic colitis submitted to total colectomy with ileostomy on D8; and lung: diffuse alveolar hemorrhage documented on D9, requiring mechanical ventilation. When the diagnosis of EGPA was made, she started intravenous pulses of methylprednisolone (1 g/day) for three days (D5-D7) followed by oral prednisolone (1 mg/kg/day).

Hospitalization was complicated by septic shock secondary to pneumonia. *Staphylococcus aureus* methicillin-resistant (MRSA), *Morganella morganii* and *Aspergillus fumigatus* were identified in bronchoalveolar lavage fluid. MRSA skin infection and *Klebsiella pneumoniae* carbapenemase-producing urinary tract infection were also documented. She did wide-spectrum antibiotics and antifungal drugs.

On D12, the infection was controlled, and the patient was hemodynamically stable, so she received intravenous immunoglobulin (2g/kg/course divided into three days). Considering the current state of infection and recent colectomy, it was decided not to perform plasma exchange nor initiate other immunosuppressive treatment. Weaning from mechanical ventilation was done, and extubation was performed on D25.

Skin lesions were getting worse over time, with more necrotic areas, deeper ulcers, and exposed bone and tendons. She underwent dressings with Dakin’s solution and was submitted to mechanical debridement of necrotic areas in the left elbow and left foot. On D32, negative pressure wound therapy was applied to the left elbow. On D33, it was decided to give another three-day course of intravenous immunoglobulin. Thirty-six days after admission to the Intensive Care Unit, the patient was transferred to the Internal Medicine Department of our hospital. At that moment, she was on prednisolone 40 mg/day.

Management in the Internal Medicine Department

**Figure 1:**
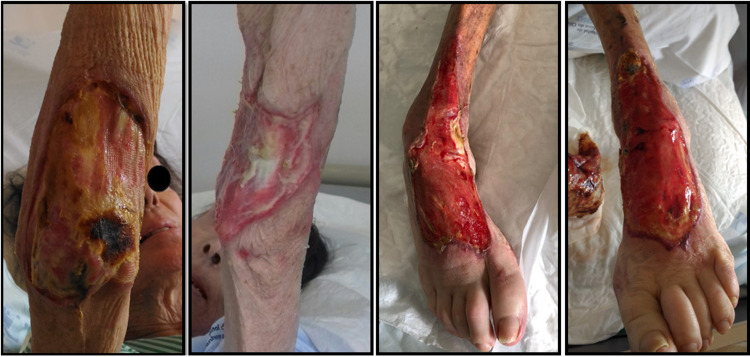
On admission to Internal Medicine Department after one month stay on Burn Intensive Care Unit Left to right: right elbow, left elbow, right foot, left foot

Upon admission, the patient’s main complaints were disabling hand and foot dysesthesias and severe loss of fine motor skills. No respiratory, gastrointestinal or urinary symptoms were present. She was hemodynamically stable, breathing room air and without fever in the last 15 days. She weighed 36.5 kg, with a body mass index of 14.26 kg/m2. She had global amyotrophy, no sitting balance and stocking-glove pattern hypoesthesia. Deep circumferential ulcers in elbows up to the muscular plane and in the dorsal side of feet with exposed bone and tendons were visible (Figure 1).

Main laboratory results at admission are shown in Table 1. Kidney function improved significantly (creatinine 0.74 mg/dL), and proteinuria was mild (662 mg/24-hour urinary sample). Screening for carbapenemase-producing *Klebsiella pneumoniae* and MRSA were positive. MRSA decolonization with mupirocin was done successfully. Chlorhexidine baths were not possible because of skin fragility and bone/tendon exposure.

**Table 1:** Main laboratory results at admission to Internal Medicine Department

Parameter		Reference value
Hemoglobin (g/dL)	11.2	12.0-16.0
White blood cells	14.8x10^3/μL	1.8-7.7
Neutrophils	5.6x10^3/μL	4.8-10.8
Eosinophils	0.2x10^3/μL	0.00-0.49
Basophils	0.0x10^3/μL	0.0-0.1
Lymphocytes	4.1x10^3/μL	1.0-4.8
Monocytes	1.1x10^3/μL	0.12-0.80
Platelets	403x10^3/μL	150-350
Erythrocyte sedimentation rate (mm/h)	68	
C-reactive protein (mg/L)	21.8	< 3.0
Urea (mg/dL)	80	15-39
Creatinine (mg/dL)	0.74	0.55-1.02
Proteins in a 24-hour urinary sample (mg/24h)	662	

According to Barthel Index, she had severe dependency for activities of daily living. She was evaluated by Clinical Nutrition and Physical Medicine and Rehabilitation and started multidisciplinary treatment with physiotherapy, occupational therapy, respiratory rehabilitation, and speech therapy.

**Figure 2:**
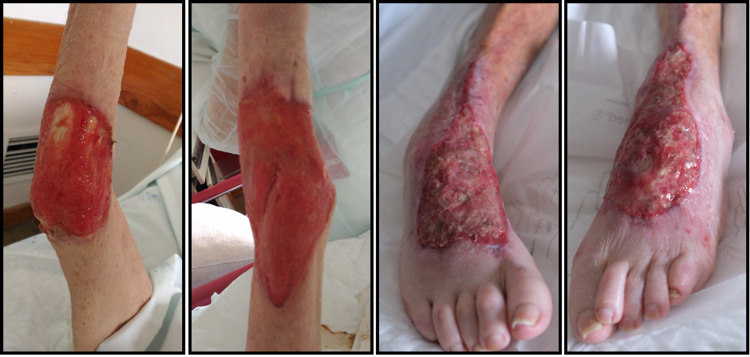
One month after admission to Internal Medicine Department, the patient maintained extensive ulcers, despite a multidisciplinary treatment approach. From left to right: right elbow, left elbow, right foot, left foot

On D11 after admission at our hospital, she developed sepsis with multiple organ failure syndrome due to a urinary tract infection complicated with *Klebsiella pneumoniae* bacteremia. She was treated with meropenem plus linezolid with good clinical response. Despite resolution of infection and all organ dysfunctions, wound dressing, adequate nutritional support, glycaemic control and rehabilitation program, she maintained a state of cachexia and did not show significant skin improvement. She had extensive circumferential ulcers in elbows and feet and kept disabling dysesthesias (Figure 2). On D33, since there was no evidence of infection and workup before starting immunosuppressive therapy was completed, we decided to start rituximab 375 mg/m2/week for four weeks. Trimethoprim-sulfamethoxazole prophylaxis was added. She was also with negative-pressure wound therapy.

**Figure 3:**
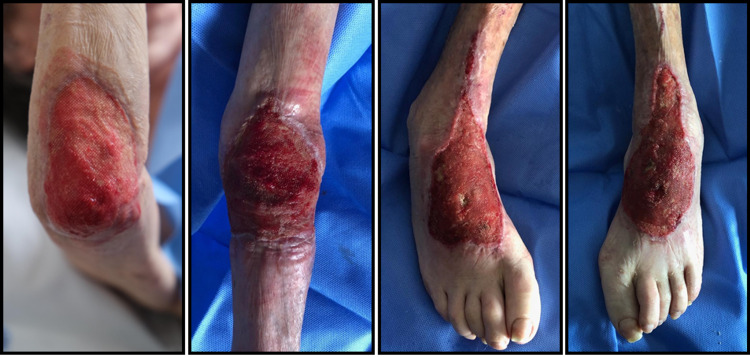
After heterologous graft repair with a regenerative dermal matrix From left to right: right elbow, left elbow, right foot, left foot

**Figure 4:**
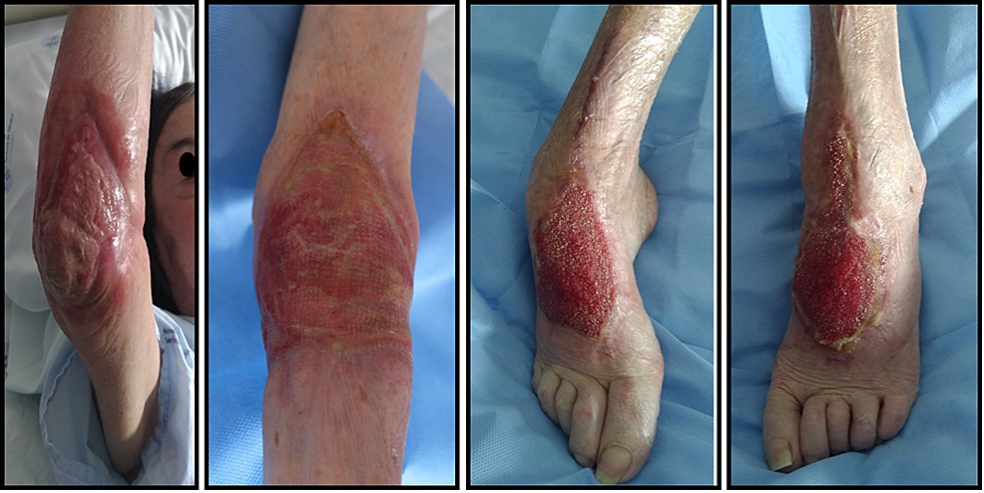
After autologous graft repair From left to right: right elbow, left elbow, right foot, left foot

The patient started to show improvements day by day. On D82, she was submitted to excisional debridement of elbows and dorsal side of feet followed by heterologous graft repair with a regenerative dermal matrix (Figure 3). The necrotic tissue was progressively substituted by granulation tissue and, later, by epithelizing tissue, with a large reduction of the wound area. Finally, on D179, she was successfully submitted to autologous graft repair of feet and elbows (Figure 4). Over time, a significant improvement of dysesthesias was noted. She started gaining weight and, after seven months of hospitalization, she weighed 50.5 kg. She progressively gained autonomy in daily activities and was able to feed on her own and help with body transfer between bed and chair. Six months after rituximab treatment, she maintained total depletion of CD20 cells. Maintenance therapy was done with azathioprine (1.5 mg/kg/day) and corticosteroid weaning was completed.

## Discussion

EGPA is a heterogeneous disease that presents with allergic and vasculitic features. Classically, three clinical stages are described: allergic stage, eosinophilic stage and vasculitic stage [4-6]. Our patient had a history of adult-onset asthma, chronic rhinitis and nasal polyposis, which correspond to the first stage. Then, eosinophilia was documented in the last year. At diagnosis, the patient presented in the vasculitic stage with severe disease and multisystem involvement.

The higher infectious risk of ANCA-associated vasculitis patients is well known [9-12]. It is related both to the disease and immunosuppressive treatment. Infection is the leading cause of death in the first year after diagnosis and a major cause of morbidity and mortality in the following years [9,11,12]. In the clinical scenario presented here, infectious complications had a great impact on the management of the patient. They narrowed the choice of immunosuppressive treatment at the beginning and, consequently, hampered the control of disease activity. On the other hand, the use of high-dose glucocorticoids possibly contributed to the several infections that the patient had and to their severity.

In the last years, advances in treatment have led to an increased rate of long-term relapse-free remission and have reduced the toxicity associated with the therapy [13]. In most trials, patients affected by EGPA were excluded. Thus, the quality of evidence for treatment management is generally lower than for other ANCA-associated vasculitides [7,8]. Up to now, for remission-induction of organ- or life-threatening disease, it is recommended a combination of glucocorticoids with either cyclophosphamide or rituximab [7,8]. In real life, rituximab is generally preferred as it is better tolerated than cyclophosphamide [14]. Our patient was severely frail and had a high infectious risk, but, at the same time, showed signs of persistent disease activity with skin ulcers, weight loss and disabling peripheral neuropathy. Therefore, the decision to start more effective immunosuppressive therapy was carefully made. After remission-induction with rituximab, the patient had a significant clinical improvement which allowed a successful graft repair.

The 2009-revised Five-Factor Score is used to evaluate prognosis at diagnosis. The following factors were significantly associated with higher five-year mortality: age ≥65 years, cardiac symptoms, gastrointestinal involvement, renal insufficiency and absence of ear-nose-throat symptoms [15]. At diagnosis, our patient had three factors of poorer prognosis (age, gastrointestinal involvement and renal insufficiency), with an estimated five-year mortality rate of 40%.

## Conclusions

EGPA is a rare and complex small-vessel vasculitis with heterogeneous clinical manifestations. Management of the disease should be patient-centered and tailored to actual clinical manifestations. We presented the case of a 68-year-old woman who came to the ED for skin lesions, which turned out to be part of an ANCA-positive EGPA with severe multisystem involvement. Simultaneously, the patient was immunocompromised and had several serious infections caused by multidrug-resistant bacteria. It was a clinical challenge to balance the need for potent immunosuppressive therapy with the high infectious risk of the patient. Nonetheless, we considered that activity disease control was fundamental to skin recovery, better physical rehabilitation and better quality of life. We would like to emphasize the importance of a multidisciplinary team working together in the approach to EGPA, where various medical areas intersect at different times and contribute to improving patient outcomes.
